# Antennal Responses and Odorant-Binding Protein 7 Binding of *Rhoptroceros cyatheae* (Selandriidae: *Rhopographus*) to Volatile Organic Compounds from *Alsophila spinulosa*

**DOI:** 10.3390/ijms27094029

**Published:** 2026-04-30

**Authors:** Mengqing Zhou, Weicheng Yang, Gaoyin Wu, Xiaona Zhang, Fen Liu, Qi Sun, Xianyu Li, Jiya Wu, Tianyu Liang, Bibo Zhou

**Affiliations:** School of Life Sciences, Guizhou Normal University, Guiyang 550025, China; zhoumengqing@gznu.edu.cn (M.Z.); wugaoyin1234@163.com (G.W.); 201606003@gznu.edu.cn (X.Z.); 15117707714@163.com (F.L.); 18096082420@163.com (Q.S.); 19306513218@163.com (X.L.); 18486593066@163.com (J.W.); 19985972222@163.com (T.L.); 19306518595@163.com (B.Z.)

**Keywords:** *Rhoptroceros cyatheae*, RcyaOBP7, host volatiles, binding characteristics, chemical ecology

## Abstract

*Rhoptroceros cyatheae* (Hymenoptera: Selandriidae) is a dominant herbivorous pest of *Alsophila spinulosa* in southwestern China, including Guizhou and Sichuan provinces. Infestation by this pest impairs spore reproduction of *A. spinulosa* and reduces the photosynthetic capacity of host plants. However, the chemosensory genes of *R. cyatheae* have not been reported, and the molecular basis of antennal detection of host volatile organic compounds (VOCs) is poorly understood. This study aims to screen and identify bioactive VOCs potentially involved in host searching behavior of *R. cyatheae*, analyze antennal VOC detection patterns, and explore the in vitro binding characteristics of an odorant-binding protein (OBP) involved in olfactory recognition, thereby providing a preliminary theoretical basis for the green management of *R. cyatheae*. Dynamic headspace sampling, gas chromatography-mass spectrometry (GC-MS), and gas chromatography-electroantennography (GC-EAD) were used to measure antennal electrophysiological responses of *R. cyatheae* to volatiles from its host *A. spinulosa.* Y-tube olfactometer assays were conducted to evaluate behavioral responses. For RcyaOBP7, fluorescence competitive binding assays, structural modeling, and molecular docking were integrated to investigate its in vitro binding characteristics with nine selected bioactive VOCs. Nine *A. spinulosa* volatiles were identified that elicited antennal electrophysiological responses in *R. cyatheae*, and the sawfly showed behavioral orientation to these VOCs, confirming that its antennae can detect host VOCs. In vitro binding assays showed that RcyaOBP7 exhibited strong binding affinity to *p*-ethylacetophenone, suggesting its potential involvement in antennal olfactory recognition of this volatile. Specific VOCs released by *A. spinulosa* are among the signaling molecules detected by the antennae of *R. cyatheae*. In vitro findings indicate that RcyaOBP7 binds specifically to *p*-ethylacetophenone, suggesting a possible role in antennal olfactory recognition and behaviors such as host location. However, in vivo functional validation and field trials under ecologically relevant conditions are needed to confirm these roles. This study characterizes the in vitro binding properties of RcyaOBP7 and provides a basis for further research on green management strategies for *R. cyatheae* based on antennal olfactory signals.

## 1. Introduction

*Alsophila spinulosa* is mainly distributed in mainland China, Southeast Asia, and southern Japan, and has existed on Earth for hundreds of millions of years [1]. It was widely distributed during the Jurassic period of the Mesozoic era and became rare in existence after the Quaternary glaciation; it is a rare and endangered relict plant categorized as a second-class national protection in China. *A. spinulosa* plays a key bridging role in the phylogenetic evolution of lower and higher plants, and has attracted widespread attention due to its multiple practical values: it not only has high ornamental value, but also serves as an important source of natural products with potential for medicinal development [2]. The components of *A. spinulosa* exhibit antibacterial, antioxidant, and antiaging properties [3,4,5]. In traditional medicine, plants of the *Alsophila* are used to treat bacterial skin infections, kidney diseases, hemorrhoids, varicose veins, diabetes, and are even employed in the preparation of tea and dressings [6]. In recent years, the larvae of *Rhoptroceros cyatheae* have extensively fed on the tender leaves of *A. spinulosa* during the sprouting period, resulting in a sharp reduction in spore production of *A. spinulosa*.

Combined with the biological characteristics of *A. spinulosa*—such as natural regeneration barriers of spores and a lengthy reproductive cycle—the reproduction and regeneration of *A. spinulosa* populations have fallen into a predicament [7,8,9,10]. Therefore, exploring the sensory recognition and host-location characteristics of *R. cyatheae* provides important theoretical support for developing targeted, eco-friendly control measures and safeguarding this endangered plant. At present, the control of the *R. cyatheae* relies mainly on physical trapping and chemical pesticides. However, the use of chemical pesticides exerts negative impacts on other organisms within the ecological system, making it imperative to develop sustainable alternative control strategies. Therefore, the development of novel biological control strategies characterized by enhanced safety, superior ecological compatibility, and minimal interference with natural ecosystems holds irreplaceable practical significance and scientific value in addressing the challenges of population regeneration in *A. spinulosa* and maintaining regional ecological balance. Semiochemicals such as insect pheromones and plant-derived volatile organic compounds (VOCs) offer a more promising alternative for pest control, characterized by species-specificity, environmental safety and benignity, as well as compatibility with integrated pest management (IPM) strategies [11,12]. Insects rely on olfaction to locate mates, food sources, and oviposition sites [13]. Previous studies have shown that eugenol, a volatile component of *Salix alba*, exhibits strong attractive activity against unmated male and female *Nematus hequensis*, and plays an important role in the host recognition of this sawfly [14]. VOCs released by plants guide herbivorous insects to locate host plants and further help them find suitable oviposition sites [15]. The development of attractants or repellents based on the interaction between insect olfaction and plant VOCs has become an important approach for green pest control [16,17]. For example, *Cephaleia chuxiongnica* shows a significantly higher tropism for volatiles from healthy pine needles than from weakened pine needles; female wasps exhibit obvious tropic responses to (−)-β-pinene, myrcene, β-caryophyllene, and other compounds, while male wasps are sensitive to (+)-α-pinene, (−)-β-pinene, and β-caryophyllene [18,19]. Female beetles show a strong tropism for buckwheat VOCs, especially prominent during the peak oviposition period, whereas male adults prefer volatiles from clover and ryegrass [20]. Previous studies have revealed that plant VOCs are closely associated with host localization of sawflies and other phytophagous insects. Nevertheless, studies concerning the attractive or repellent responses of *R. cyatheae* to volatiles from *A. spinulosa* remain unclear.

The recognition and detection of VOCs by insects are mainly driven by odorant-binding proteins (OBPs), which can specifically bind and transport odor molecules, thereby triggering downstream signal transduction and behavioral responses [21]. Although the number of OBPs is far lower than the variety of volatile compounds in nature, most OBPs exhibit broad-spectrum binding properties and can recognize and bind to multiple odor ligands [22,23]. In addition to participating in olfactory recognition, OBPs have also been reported to be involved in non-olfactory physiological processes such as taste perception, humidity sensing, immune response, and flight behavior [24,25,26,27], making them important potential targets for the development of behavior-modulating agents of agricultural and forestry pests [28]. Therefore, investigating the binding characteristics between OBPs of *R. cyatheae* and host volatiles is crucial for revealing their chemical ecological adaptation characteristics and developing green control strategies based on development.

Until now, the lack of systematic research on the molecular basis of olfactory detection in *R. cyatheae* has greatly limited the development of targeted eco-friendly management strategies. In previous work by our group, OBP7 was mined from *R. cyatheae* using transcriptome data, and RcyaOBP7 was abundantly expressed in the antennae of both male and female individuals. Data on RcyaOBP7 were obtained from our transcriptome analysis (submitted for publication). On this basis, this study combines electrophysiological detection, behavioral bioassays, recombinant protein expression and purification, fluorescence competitive binding assays, and molecular docking to explore the binding profiles between host plant *A. spinulosa* VOCs and RcyaOBP7, and identify semiochemical candidates for the sustainable management of *R. cyatheae*. Findings from this study seek to provide theoretical foundations and technical references for the development of novel insect behavior-modulating agents (attractants/repellents) and the subsequent characterization of olfactory receptor ligands of *R. cyatheae*, help reduce the application of chemical pesticides, improve the natural regeneration and viability of *A. spinulosa* populations, and ultimately offer new strategies for the protection of this rare and endangered relict plant and the green control of its pests.

## 2. Results

### 2.1. Components of A. spinulosa and GC-EAD

Volatile components of *A. spinulosa* were collected by headspace dynamic adsorption, and separated and identified using GC-MS. During the experiment, the RI of individual compounds was calculated with a reference mixture of n-alkanes (C_6_–C_30_), combined with NIST mass spectral library matching, RI comparison, and analysis of mass spectral fragmentation patterns. A total of 39 volatile compounds were ultimately identified from the volatiles of *A. spinulosa*, covering various classes, including alkenes, alcohols, alkanes, ketones, esters, and aromatic compounds; detailed compositional data are listed in Table 1. Among these compounds, 2-ethyl-1-hexanol was the most abundant, accounting for 24.60%, followed by *p*-ethylacetophenone (22.74%). Other major components were 3-ethyl-2-hexene (6.64%), tridecane (6.51%), thymol (5.29%), 2-methyl-2-heptene (4.30%), 3-methylheptane (4.06%), and others.

**Figure 1:**
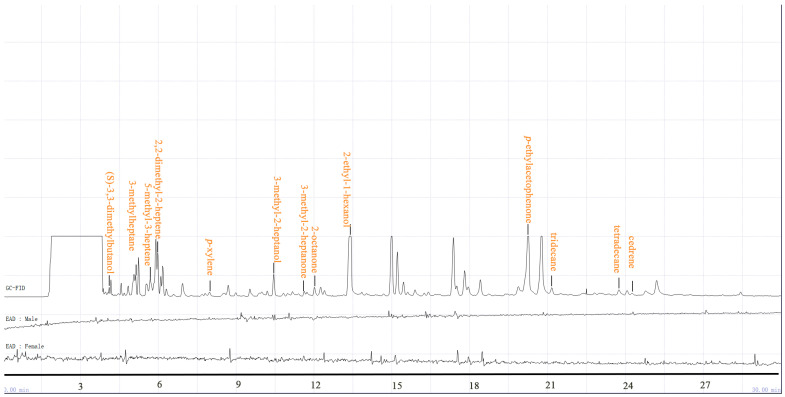
GC-EAD detection of the responses of *R. cyatheae* to volatiles from *A. spinulosa*.

After repeated tests and comparative analyses via GC-EAD, the GC-EAD responses of male and female (1–3 days post-emergence, reared separately to ensure unmated status) antennae of the *R. cyatheae* to the extracted volatile components of *A. spinulosa* were recorded, and the results are displayed in Figure 1. A total of 13 electrophysiologically active compounds in the volatile samples from *A.spinulosa* elicited EAG responses in *R. cyatheae*. Based on the retention times and mass spectral fingerprints obtained via GC-MS, combined with the screening results of EAD activity, EAD-active compounds were successfully identified through the comparative analysis of characteristic data. These electrophysiologically active compounds are as follows: (S)-3,3-dimethyl-2-butanol, 3-methylheptane, *p*-xylene, 2,2-dimethyl-2-heptene, 5-Methyl-3-heptene, 3-methyl-2-heptanol, 3-methyl-2-heptanone, 2-octanone, 2-ethyl-1-hexanol, *p*-ethylacetophenone, tridecane, tetradecane and cedrene (Table 2).

### 2.2. Electrophysiological Responses of Male and Female R. cyatheae to Nine VOCs

**Figure 2:**
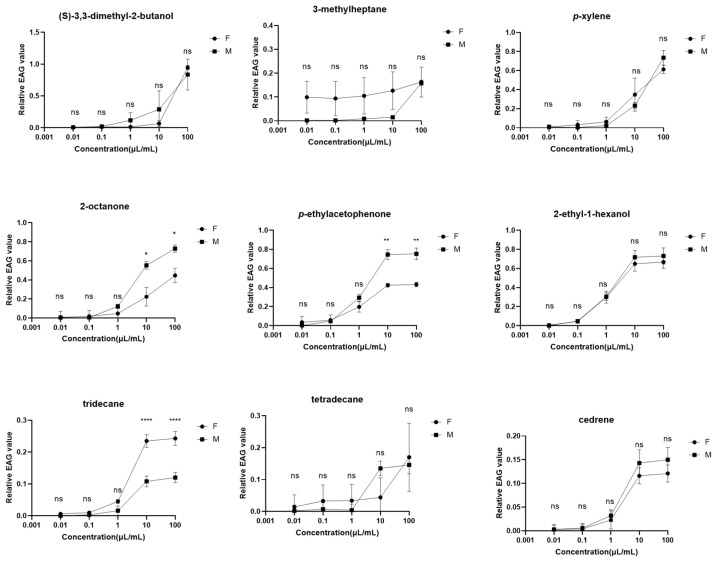
EAG response curves corresponding to concentration gradients of 9 VOCs (mean ± standard error, SE; *n* = 3). Data represent the mean values of three replicate experiments; error bars indicate the standard error (SE) of the means. * indicates significant differences between white bars and black bars (* *p* < 0.05; ** *p* < 0.01; **** *p* < 0.0001). ns: not significant.

Through comparative analysis of the bioactive VOCs from *A. spinulosa* plants by GC-EAD, this study selected 9 commercially available VOCs with qualified purity and stable physicochemical properties for EAG assays. Constrained by the absence of commercially available high-purity standards, high synthetic difficulty, or insufficient stability of certain target volatiles, these compounds failed to meet the stringent requirements of electrophysiological experiments. Consequently, EAG responses of the antennae of *R. cyatheae* adults were only measured for the above 9 VOCs. The relative EAG values of the antennae of male and female adult sawflies in response to (S)-3,3-dimethyl-2-butanol, 3-methylheptane, *p*-xylene, 2-octanone, 2-ethyl-1-hexanol, *p*-ethylacetophenone, tridecane, tetradecane, and cedrene are shown in Figure 2 and Table S1. This study investigated the effects of three factors, i.e., concentration gradient, sexual dimorphism, and VOC species, on the relative EAG values of *A. spinulosa*, with the results detailed in the following.

The overall effect of concentration gradient on the relative EAG values of male and female adult sawflies manifested as a general upward trend in relative EAG values with increasing VOC concentrations. For most VOCs (including *p*-xylene, (S)-3,3-dimethyl-2-butanol, *p*-ethylacetophenone, and 2-ethyl-1-hexanol), their relative EAG values in sawflies of the same sex exhibited a significant concentration-dependent increase as the VOC concentration rose from 0.01 μL/mL to 100 μL/mL. In the low-concentration range (0.01 and 0.1 μL/mL), the relative EAG values induced by all tested VOCs were generally low, mostly below 0.1 mV. For example, the relative EAG values of both male and female sawflies exposed to *p*-xylene at 0.01 μL/mL were lower than 0.01 mV. In the high-concentration range (10 and 100 μL/mL), by contrast, the relative EAG values of sawflies were significantly elevated relative to the low-concentration groups, and the response peaks of some VOCs showed a pronounced upward trend in this concentration range. Overall, the relative EAG values induced by most VOCs displayed evident concentration dependence with changing concentrations.

Sexual differences existed in the relative EAG values of male and female adult sawflies, and such differences were concentration-dependent. No significant sexual differences were observed in the relative EAG values elicited by all tested VOCs in the low-concentration range (0.01 and 0.1 μL/mL) and at 10 μL/mL. However, the relative EAG values induced by several VOCs differed significantly between males and females in the high-concentration range. Specifically, 2-octanone induced significant sexual differences in relative EAG values at 10 μL/mL and 100 μL/mL, and tridecane caused highly significant sexual differences, whereas no significant sexual differences were detected for the other VOCs in this concentration range.

### 2.3. Behavioral Responses of Male and Female R. cyatheae to Nine Active VOCs

**Figure 3:**
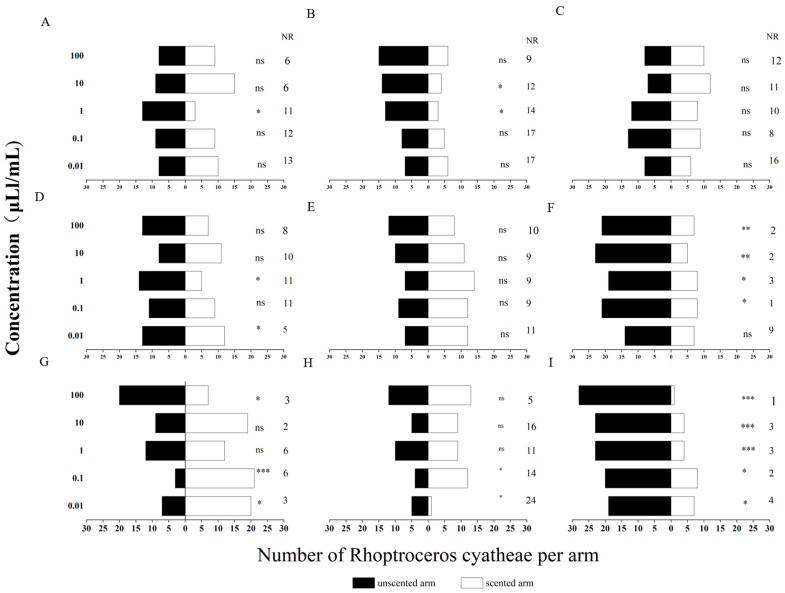
Behavioral responses of male adults of *R. cyatheae* to tridecane (**A**), tetradecane (**B**), *p*-xylene (**C**), *p*-ethylacetophenone (**D**), cedrene (**E**), 3-methylheptane (**F**), 2-octanone (**G**), 2-ethyl-1-hexanol (**H**), and (S)-3,3-dimethyl-2-butanol (**I**). * indicates significant differences between white bars and black bars (* *p* < 0.05; ** *p* < 0.01; *** *p* < 0.001). NR: percentage of non-responders. ns: not significant.

**Figure 4:**
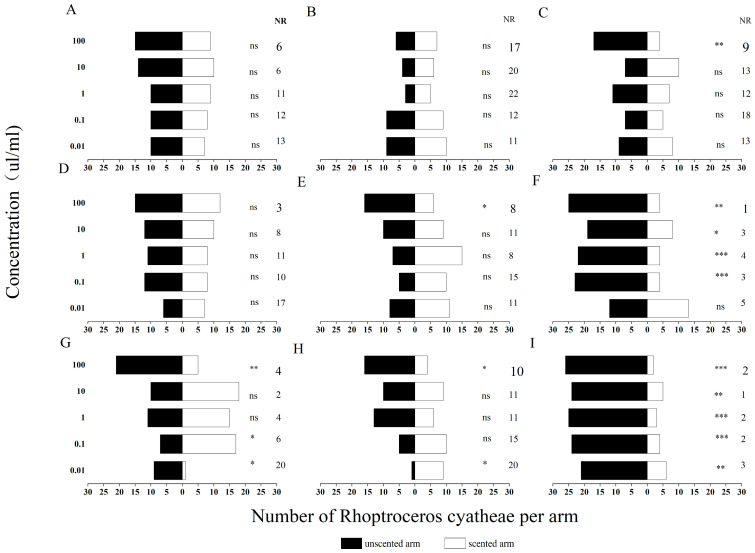
Behavioral responses of female adults of *R. cyatheae* to tridecane (**A**), tetradecane (**B**), *p*-xylene (**C**), *p*-ethylacetophenone (**D**), cedrene (**E**), 3-methylheptane (**F**), 2-octanone (**G**), 2-ethyl-1-hexanol (**H**), and (S)-3,3-dimethyl-2-butanol (**I**). * indicates significant differences between white bars and black bars (* *p* < 0.05; ** *p* < 0.01; *** *p* < 0.001). NR: percentage of non-responders. ns: not significant.

In this study, a two-arm Y-tube olfactometer was used to determine the behavioral responses of unmated male and female adult *R. cyatheae* to host plant volatiles, and a chi-square (χ^2^) test was conducted to analyze the differences in responses of test insects to the unscented arm and the scented arm of the same sex and at the same concentration. At the experimental concentrations (0.01, 0.1, 1, 10, 100 μL/mL), the behavioral responses of male and female *R. cyatheae* to nine VOCs, namely (S)-3,3-dimethyl-2-butanol, 2-octanone, 3-methylheptane, *p*-xylene, *p*-ethylacetophenone, 2-ethyl-1-hexanol, cedrene, tridecane, and tetradecane, were assayed (Figure 3 and Figure 4).

Behavioral responses of male and female R. cyatheae to nine VOCs were determined at five concentration gradients.. As the concentration increased, the selection preference of unmated *R. cyatheae* gradually increased. At 0.01 μL/mL, a significant attraction of females to 2-ethyl-1-hexanol was observed (χ^2^ = 6.400, df = 1, *p* < 0.05). In contrast, females showed a significant repellent response to 2-octanone (χ^2^ = 6.259, df = 1, *p* < 0.05), while males were significantly attracted to 2-octanone (χ^2^ = 6.400, df = 1, *p* < 0.05). Both females and males exhibited significant repellent responses to (S)-3,3-dimethyl-2-butanol (females: χ^2^ = 8.333, df = 1, *p* < 0.01; males: χ^2^ = 5.538, df = 1, *p* < 0.05).

At 0.1 μL/mL, male sawflies exhibited a significant repellent response to 2-octanone (χ^2^ = 4.000, df = 1, *p* < 0.05); both male and female sawflies showed a significant attractive response to *p*-ethylacetophenone (χ^2^ = 4.167, df = 1, *p* < 0.05; χ^2^ = 13.500, df = 1, *p* < 0.001); both sexes displayed a significant repellent response to 3-methylheptane (χ^2^ = 13.370, df = 1, *p* < 0.001; χ^2^ = 5.828, df = 1, *p* < 0.05); and both sexes exhibited a significant repellent response to (S)-3,3-dimethyl-2-butanol (χ^2^ = 14.286, df = 1, *p* < 0.001; χ^2^ = 5.134, df = 1, *p* < 0.05).

At 1 μL/mL, males showed a significant repellent response to *p*-ethylacetophenone (χ^2^ = 4.263, df = 1, *p* < 0.05). Both females and males exhibited significant repellent responses to 3-methylheptane (females: χ^2^ = 12.462, df = 1, *p* < 0.001; males: χ^2^ = 4.481, df = 1, *p* < 0.05). Furthermore, a highly significant repellent effect of (S)-3,3-dimethyl-2-butanol was observed in both sexes (females: χ^2^ = 17.286, df = 1, *p* < 0.001; males: χ^2^ = 13.370, df = 1, *p* < 0.001). Males also displayed significant repellent responses to tridecane and tetradecane (tridecane: χ^2^ = 6.250, df = 1, *p* < 0.05; tetradecane: χ^2^ = 6.250, df = 1, *p* < 0.05).

At 10 μL/mL, both females and males showed a significant repellent response to 3-methylheptane (females: χ^2^ = 4.481, df = 1, *p* < 0.05; males: χ^2^ = 11.571, df = 1, *p* < 0.01). Additionally, a highly significant repellent effect of (S)-3,3-dimethyl-2-butanol was observed in both sexes (females: χ^2^ = 13.370, df = 1, *p* < 0.001; males: χ^2^ = 12.448, df = 1, *p* < 0.001). Males also exhibited a significant repellent response to tetradecane (χ^2^ = 5.556, df = 1, *p* < 0.05).

At 100 μL/mL, female sawflies exhibited a significant repellent response to 2-ethyl-1-hexanol (χ^2^ = 7.200, df = 1, *p* < 0.01); both male and female sawflies showed a significant repellent response to 2-octanone (χ^2^ = 9.846, df = 1, *p* < 0.01; χ^2^ = 6.259, df = 1, *p* < 0.05); both sexes displayed a significant repellent response to 3-methylheptane (χ^2^ = 15.207, df = 1, *p* < 0.001; χ^2^ = 7.000, df = 1, *p* < 0.01); both sexes had a highly significant repellent response to (S)-3,3-dimethyl-2-butanol (χ^2^ = 20.571, df = 1, *p* < 0.001; χ^2^ = 25.138, df = 1, *p* < 0.001); and both sexes showed a significant repellent response to cedrene and *p*-xylene (Cedrene: χ^2^ = 4.545, df = 1, *p* < 0.05; *p*-xylene: χ^2^ = 8.048, df = 1, *p* < 0.05).

Additionally, 2-ethyl-1-hexanol and 2-octanone induced significant attractive responses at low concentrations but significant repellent responses at high concentrations. Changes in concentration had little effect on 3-methylheptane and (S)-3,3-dimethyl-2-butanol, with sawflies exhibiting a consistent repellent response to both compounds across all concentration gradients. The other VOCs failed to elicit any significant repellent or attractive responses from sawflies as the concentration varied. All nine of these VOCs were subsequently used for the follow-up fluorescence competitive binding assays.

### 2.4. Expression and Purification of Recombinant RcyaOBP7

**Figure 5:**
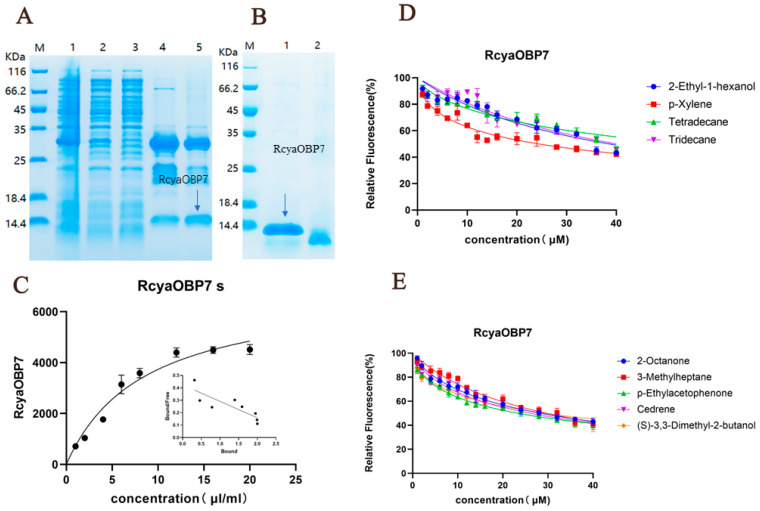
SDS-PAGE (**A**,**B**) of RcyaOBP7. Lane M: Protein Marker; Lane 1: Precipitate after lysis; Lane 2: Supernatant after lysis; Lane 3: Effluent; Lane 4: Washing sample; Lane 5: Elution sample. Right of Lane M: Protein Marker; Lane 1: Reduced sample; Lane 2: Non-reduced sample. Arrows indicate the target RcyaOBP7 protein bands. Ligand binding assays of the RcyaOBP7 (**C**) Binding curves and relative Scatchard plots (insert) of RcyaOBP7 with fluorescentprobe1-NPN (**C**) Competitive binding curves of selected ligands to the RcyaOBP7. The ligand names are shown to the right of the curves (**D**,**E**).

Recombinant RcyaOBP7 was successfully induced and heterologously expressed in *Escherichia coli* BL21, and its expression was verified by SDS-PAGE and Western blot analysis (Figure S3). After purification (Figure 5A), the His-tag was cleaved using SUMO protease. The concentration of the purified RcyaOBP7 protein was determined to be 1.04 mg/mL. Reducing and non-reducing SDS-PAGE analysis confirmed that the protein achieved high purity (Figure 5B), showed normal folding without abnormal aggregation, and thus met the requirements for subsequent experiments.

### 2.5. Binding Affinity of RcyaOBP7

To evaluate the binding affinity of RcyaOBP7, fluorescence competitive binding assays were performed using nine candidate ligands. The dissociation constant (K_1_-NPN) between RcyaOBP7 and the fluorescent probe 1-NPN was measured as 8.465 μM. A linear Scatchard plot was obtained, indicating that 1-NPN is a reliable probe (Figure 5C).

**Figure 6:**
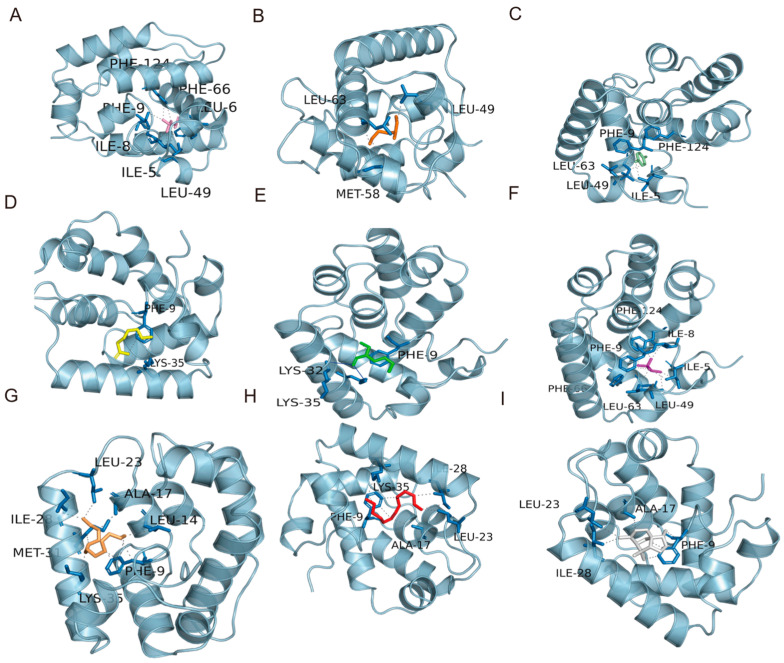
Molecular docking of RcyaOBP7 to ligands. The blue solid lines represent hydrogen bonds between ligands and RcyaOBP7, while the grey dashed lines represent hydrophobic interactions. The protein structure is shown in cyan, and different ligands are marked with distinct colors. (S)-3,3-dimethyl-2-butanol (**A**), 3-methylheptane (**B**), *p*-xylene (**C**), 2-octanone (**D**), 2-ethyl-1-hexanol (**E**), *p*-ethylacetophenone (**F**), tridecane (**G**), tetradecane (**H**), and cedrene (**I**).

Using 1-NPN as the fluorescent probe, the competitive binding affinities of RcyaOBP7 for nine VOCs from *A. spinulosa* were determined. The half-maximal inhibitory concentration (IC_50_) and inhibition constant (Ki) were calculated from the binding curves (Figure 5D,E). As shown in Table 3, one of the nine ligands could displace 1-NPN from the RcyaOBP7/1-NPN complex. The results demonstrated that RcyaOBP7 exhibited strong binding affinity toward *p*-ethylacetophenone at pH 7.4, with a Ki value of 18.12 μmol/L. The binding energy of this volatile compound was −6.1 kcal/mol (Figure 6; Table 3).

### 2.6. Structural and Docking Analysis Results

The three-dimensional structure of RcyaOBP7 was obtained by homology modeling using Robetta. Ramachandran plot analysis was performed with the Procheck program to evaluate the structure quality. The results showed that 93.6% and 5.6% of the amino acid residues in RcyaOBP7 were located in the most favored regions and additionally allowed regions, respectively; no residues were found in the generously allowed regions, and only 0.8% were present in the disallowed regions (Figure S1). These results confirmed that the constructed protein structure was reliable and suitable for subsequent analysis. In this study, molecular docking was performed between RcyaOBP7 of *R. cyatheae* and the test VOCs. The binding strength between the protein and each compound was assessed by binding energy: a more negative value indicates a stronger protein–ligand interaction.

To further explore the binding characteristics of the RcyaOBP7 protein, molecular docking was used to analyze the binding free energy and key binding amino acid residues between RcyaOBP7 and its ligands. RcyaOBP7 could bind to eight candidate ligands, with binding free energies ranging from −4.30 to −7.10 kcal/mol (Table 3). Among the tested compounds, cedrene exhibited the strongest binding affinity to RcyaOBP7, with a binding energy of −7.1 kcal/mol, followed by *p*-ethylacetophenone with a binding energy of −6.1 kcal/mol, and then *p*-xylene with a binding energy of −5.9 kcal/mol. The VOCs (S)-3,3-dimethyl-2-butanol, 3-methylheptane, 2-octanone, 2-ethyl-1-hexanol,tridecane, and tetradecane all showed binding energies of less than −5.0 kcal/mol with RcyaOBP7. However, (S)-3,3-dimethyl-2-butanol, 3-methylheptane, and 2-octanone displayed low binding affinity to RcyaOBP7, even though they elicited strong responses in both EAG and behavioral assays. 2-ethyl-1-hexanol and tridecane showed the lowest binding energies among all ligands (Table 3); meanwhile, these two ligands did not have the lowest inhibition constants (Ki values) among the nine tested ligands. These results indicated that molecular docking can effectively predict the protein–ligand binding characteristics to a certain extent. Specifically, *p*-ethylacetophenone, cedrene, and *p*-xylene formed the strongest interactions with RcyaOBP7. The binding of *p*-ethylacetophenone to the protein was mediated solely by hydrophobic interactions, involving PHE9, PHE66, PHE124, LEU49, LEU63, ILE5, and ILE8. The binding of Cedrene was also mediated by hydrophobic interactions, with PHE9, ILE23, ILE28, and ALA17 participating (Figure 6). Similarly, the binding of *p*-xylene was mediated exclusively by hydrophobic interactions, involving PHE9, PHE124, LEU49, LEU63, and ILE5 (Figure 6).

**Table 3:** Binding affinities of RcyaOBP7 recombinant proteins for 9 ligands identified from *A. spinulosa* volatiles.

		RcyaOBP7		
No	Ligands	IC50 (μM)	Ki (μM)	Binding Energy (kcal/mol)
1	*p*-ethylacetophenone	19.58 ± 0.91	18.12 ± 1.83	−6.1
2	*p*-xylene	24.36 ± 2.36	22.54 ± 2.18	−5.9
3	(S)-3,3-dimethyl-2-butanol	31.29 ± 2.97	28.96 ± 2.35	−4.9
4	3-methylheptane	40.99 ± 5.60	37.94 ± 5.37	−4.6
5	2-octanone	28.34 ± 1.83	26.23 ± 1.80	−4.4
6	cedrene	46.23 ± 6.00	42.79 ± 5.51	−7.1
7	tridecane	46.20 ± 4.71	42.76 ± 4.41	−4.3
8	tetradecane	44.02 ± 4.98	40.75 ± 4.61	−4.6
9	2-ethyl-1-hexanol	38.57 ± 3.10	35.70 ± 2.87	−4.3

Note: Data are presented as “mean”. The binding affinity for Ki > 40 μM is considered weak.

## 3. Discussion

Antenna-specifically expressed OBPs are involved in volatile signal detection, and elucidating their functions contributes to understanding olfactory processes in insects. Focusing on the olfactory responses of *R. cyatheae* to its host plant *A. spinulosa*, this study addresses a research gap in the chemical ecological interactions between a rare and endangered plant and its specialist pest, offering a perspective for the conservation of *A. spinulosa* resources and the potential green control of *R. cyatheae*. This study employed headspace dynamic adsorption to collect volatile components from *A. spinulosa*, followed by separation and identification using GC-MS. Compounds were identified through NIST spectral library matching, RI comparison, and analysis of mass spectral fragmentation patterns. Retention indices were calculated using a series of n-alkane standards (C_6_–C_30_). A total of 39 volatile compounds were detected from *A. spinulosa*, including alkenes, alcohols, alkanes, ketones, esters, and aromatic compounds (Table 1). Among these, 2-ethyl-1-hexanol was the most abundant component (24.60%), followed by *p*-ethylacetophenone. Other major components included 3-ethyl-2-hexene (6.44%), tridecane (6.51%), thymol (5.29%), 2-methyl-2-heptene (4.30%), and 3-methylheptane (4.06%). GC-EAD identified 13 VOCs that exhibited electrophysiological activity on the antennae of the *R. cyatheae*, of which nine were commercially available. Further validation using EAG and behavioral assays indicated attractant or repellent effects of these compounds on the sawfly.

Based on antennal transcriptome data (Supplementary Materials), the antenna-enriched gene RcyaOBP7 was selected. Following prokaryotic expression and protein purification, fluorescence competitive binding assays were performed between the recombinant protein and the nine VOCs. This study characterized the in vitro binding properties of RcyaOBP7 with *A. spinulosa* volatiles, contributing to the understanding of antennal olfactory recognition in *Rhopographus* insects. Binding affinity analysis showed that RcyaOBP7 exhibited strong binding capacity to *p*-ethylacetophenone in vitro. Homology modeling and molecular docking were also used to complement the binding affinity data. However, this study focused on a single OBP gene (RcyaOBP7) and did not investigate the expression patterns or functions of other OBP genes in the antennae of *R. cyatheae*. Therefore, the synergistic effects of the OBP family in antennal olfactory perception remain unclear. Future studies should explore synergistic interactions among different OBPs and other olfactory-related genes to further clarify the molecular basis of antennal olfactory recognition in this sawfly.

EAG and behavioral assays showed that virgin male and female adults of *R. cyatheae* exhibited concentration-dependent olfactory responses to host plant volatiles. Two alcoholic compounds, (S)-3,3-dimethyl-2-butanol and 2-ethyl-1-hexanol, induced relatively strong EAG responses in both sexes, suggesting that these compounds may be behaviorally active for olfactory neurons of *R. cyatheae.* The study further found that (S)-3,3-dimethyl-2-butanol triggered significant EAG responses and repellent effects in both sexes. To date, no studies have directly reported the effects of (S)-3,3-dimethyl-2-butanol on insect behavioral responses. The structural moiety of this compound has been used in the synthesis of various bioactive compounds, including neuroactive 2,4-dicarboxypyrrole mGluR1 antagonists [29] and azole derivatives with antifungal activities [30]. These findings suggest potential chemosensory functions of this compound and its structural analogs in insects. Notably, fluorescence competitive binding assays and molecular docking showed that RcyaOBP7 exhibited only moderate binding affinity to (S)-3,3-dimethyl-2-butanol, with no stable intermolecular interactions detected. Nevertheless, (S)-3,3-dimethyl-2-butanol shows promise as an effective repellent for *R. cyatheae* and warrants further evaluation in the development of insect behavioral modulators.

EAG responses of virgin male and female *R. cyatheae* to 2-ethyl-1-hexanol increased in a concentration-dependent manner. Previous studies have reported that 2-ethyl-1-hexanol is a volatile released by maize and rice, with attractive activity against two weevil species: *Sitophilus zeamais* shows tropism at relatively low concentrations, whereas *S. oryzae* exhibits significant responses only at higher concentrations [31]. In *Papilio xuthus*, the emission of this compound changes before and after mating, suggesting a possible role in mate recognition [32]. It is also an EAG-active component in host-related odors of parasitoid wasps and has been implicated in tritrophic interactions [33]. In the present study, Y-tube olfactometer assays showed that virgin *R. cyatheae* adults could discriminate host plant VOCs and exhibited sex-specific olfactory behaviors, with virgin males showing strong attraction to 2-ethyl-1-hexanol. Similar sex-specific responses have been observed in other insects: *Trabala vishnou* females are attracted while males show no obvious response [34]; in *Leucinodes orbonalis*, the attraction index of this compound is higher for females than males [35]. Field trials against *Hypsipyla robusta* showed that blended attractants containing this compound increased trapping efficiency by 40% compared with single compounds [36]. In Diptera, 2-ethyl-1-hexanol enhanced midge taxis toward CO_2_, improving attraction by 35% [37], and it participates in host plant recognition by *Sitodiplosis mosellana* [38]. In Hymenoptera, this compound is also involved in host location by parasitoid wasps [33].

Besides alcohols, the ketone compound *p*-ethylacetophenone elicited strong EAG responses in males, while females exhibited more significant EAG responses to 2-octanone. *p*-xylene induced moderate-to-high EAG responses in both sexes. As for *p*-ethylacetophenone, virgin male and female *R. cyatheae* showed significantly stronger preferences for this compound with increasing concentration. In other insect species, female *Anoplophora glabripennis* exhibited significantly higher sensitivity to this compound than males, which is associated with their biological need to locate suitable oviposition sites [39]. *p*-Ethylacetophenone is also one of the key volatiles mediating host recognition in *Agrilus lewisiellus* and *Megastigmus sabinae*, regulating adult feeding and oviposition site localization [40,41]. In addition, when blended with β-ocimene, the attraction index of this compound was remarkably higher than that of the single compound, showing a significant synergistic attractive effect [42]. 2-Octanone is a volatile compound widely distributed in plants, insects, their excreta, and some microorganisms [43,44,45]. In *R. cyatheae*, virgin males and females displayed a strong positive preference for low concentrations of 2-octanone, while shifting to a negative preference (repellent effect) at high concentrations, showing a typical pattern of attraction at low concentrations and repellency at high concentrations. Previous studies found that 2-octanone in the excreta of *Cimex hemipterus* only attracted males [46]. 2-Octanone showed weak attractive activity when used alone, but its attractive effect was significantly enhanced after blending with other yeast volatiles, especially in regulating oviposition site selection of females [47]. When combined with host volatiles such as linalool and limonene, it could significantly improve the attractive activity against *Bactrocera oleae* [48]. Yeast fermentation products containing 2-octanone exhibited significantly better attractiveness to *B. oleae* than traditional protein baits, reducing chemical pesticide application by 30–40% [49]. Aromatic hydrocarbons ranked next. In this study, *p*-xylene elicited weak EAG responses in both male and female sawflies. As a “signal modulator” in plant volatile mixtures, it mediates insect host recognition synergistically with other components. Virgin females displayed significant negative preference and obvious repellency to high concentrations of *p*-xylene, which is consistent with its repellent effect on adults of *Plutella xylostella* [50]. Although *p*-xylene showed no significant attractive or repellent activity to male and female adults of *Phauda flammans*, blending it with fig volatiles such as benzaldehyde and acetophenone could improve the overall attractive effect and regulate insect host selection behavior [51].

In contrast, alkane compounds including 3-methylheptane, tridecane, tetradecane and cedrene only elicited weak EAG responses in both male and female sawflies. This study further revealed that the differences in relative EAG responses between male and female *R. cyatheae* were concentration-dependent, with more prominent disparities under high concentrations. For most volatile compounds, male sawflies exhibited stronger responses than females. On the whole, the relative EAG response values presented an upward trend along with the increase in the concentration of tested compounds. 3-methylheptane showed slightly higher EAG activity in females than in males, but its overall activity was weak. Relevant studies have demonstrated that blending 3-methylheptane with *p*-ethylacetophenone in proper proportions can increase the attraction rate by approximately 50% and promote insect oviposition, showing a remarkable synergistic attractive effect [52]. Females exhibited great individual variation in EAG responses to low concentrations of cedrene, but no obvious behavioral response was induced. This phenomenon is mainly attributed to two factors: first, the sensitivity threshold of EAG detection is lower than the threshold for behavioral responses; second, olfactory adaptation caused by peripheral receptor desensitization and central nervous system regulation in insects [53].

EAG and Y-tube olfactometer assays demonstrated that virgin male and female *R. cyatheae* could distinguish VOCs from host plants and displayed sex-specific olfactory responses. Males exhibited stronger EAG responses to most tested compounds than females, with more pronounced sexual dimorphism observed at higher concentrations. These findings identify (S)-3,3-dimethyl-2-butanol, 2-ethyl-1-hexanol, *p*-ethylacetophenone, and 2-octanone as EAG-active compounds for *R. cyatheae*, highlighting their potential application in semiochemical-based pest monitoring strategies. Notably, the Y-tube olfactometer experiments were performed under laboratory conditions, where the volatile concentrations employed (e.g., 1 μL/mL) may exceed those naturally emitted by host plants. Furthermore, host plants in natural settings release complex volatile blends rather than individual compounds; thus, the attractant or repellent effects of individual compounds observed in the laboratory cannot be directly extrapolated to natural ecosystems. Additionally, this study only assessed orientation behavior via Y-tube olfactometers, which may not fully capture the olfactory perception and behavioral responses of *R. cyatheae* in natural environments. Future research should integrate field trials to validate the roles of key VOCs and RcyaOBP7 in the olfactory perception and host location of *R. cyatheae* under ecologically relevant conditions, thereby providing more reliable theoretical support for the development of green pest control technologies.

Based on these results, it is possible that some members of the RcyaOBP family in *R. cyatheae* may be involved in host localization by binding to volatiles from *A. spinulosa*. Recombinant RcyaOBP7 showed differential binding affinities toward nine volatiles (Table 4): strong binding to *p*-ethylacetophenone, moderate binding to 2-octanone, 2-ethyl-1-hexanol, *p*-xylene, (S)-3,3-dimethyl-2-butanol, and 3-methylheptane, and weak binding to tridecane, tetradecane, and cedrene. Molecular docking results indicated favorable binding of RcyaOBP7 to cedrene, *p*-ethylacetophenone, and *p*-xylene. Hydrophobic interactions and aromatic residues were important for ligand binding [54], and all intermolecular forces between the nine ligands and RcyaOBP7 were hydrophobic in nature. *p*-Ethylacetophenone, cedrene, and *p*-xylene formed stable binding conformations, while the other compounds formed unstable conformations. *p*-Ethylacetophenone is a volatile component of numerous plants, including *cotton*, *Ocimum species*, *tea plants*, and *Passiflora incarnata* [55,56,57,58]. 2-Octanone is widely distributed in plants and can be emitted through normal metabolism or induced by insect herbivory [59,60,61,62]. *p*-Xylene is an aromatic hydrocarbon in floral volatiles of *Syringa* plants [63,64], and shows moderate binding affinity to GdauOBP20 from *Galeruca daurica* and OBPs from *Adelphocoris lineolatus* [65,66]. Its emission increases significantly in plants under herbivory, mechanical wounding, or environmental stress, suggesting a possible role in indirect plant defense [55,67,68]. 3-Methylheptane has been reported in various plants, including *Ligularia virgaurea* [69]. Future studies should verify the behavioral effects of these volatiles on *R. cyatheae* under ecologically relevant conditions.

In the present study, *R. cyatheae* sawflies exhibited relatively high EAG responses to *p*-ethylacetophenone. Behavioral assays revealed that male sawflies displayed obvious repellent effects at the concentration of 1 μL/mL. Combined with the results of fluorescence competitive binding assays and molecular docking, RcyaOBP7 showed strong interactions with *p*-ethylacetophenone, which were dominated by hydrophobic interactions. The key binding amino acid residues were identified as His9, Phe66, Phe124, Leu49, Leu63, Ile5 and Ile8. (S)-3,3-dimethyl-2-butanol, 2-ethyl-1-hexanol, *p*-xylene and 2-octanone induced relatively strong EAG responses in sawflies, whereas 3-methylheptane, tridecane, tetradecane and cedrene only triggered weak EAG reactions. Nevertheless, behavioral tests demonstrated distinct sexual differences in insect responses to these volatiles: (S)-3,3-dimethyl-2-butanol exerted remarkable repellent effects; 2-ethyl-1-hexanol showed attractive effects at low concentrations and repellent effects at high concentrations; 2-octanone exhibited significant attractive activity; *p*-xylene caused no obvious behavioral responses; 3-methylheptane induced prominent repellency; and tridecane, tetradecane, as well as cedrene, showed no significant attractive or repellent effects on the sawflies. Except for *p*-ethylacetophenone, the other six VOCs exhibited weak binding affinity to RcyaOBP7 and poor binding performance in molecular docking. Collectively, our results reveal a clear decoupling among OBP binding affinity, in vitro molecular interaction, peripheral EAG detection, and final behavioral output. Such inconsistencies are not caused by experimental artifacts but represent intrinsic properties of insect odorant recognition. Although structural incompatibility and steric hindrance within the binding pocket partially explain this mismatch [70], multiple synergistic regulatory mechanisms at peripheral and neural levels should not be overlooked: First, alternative OBPs or chemosensory proteins (CSPs) may mediate the transportation and delivery of these volatiles [71]. Second, odorant receptors (ORs) can recognize semiochemicals through OBP-independent signaling cascades [72]. Third, peripheral olfactory detection cannot fully represent downstream neural regulation, and higher-order integration in the central nervous system further reshapes behavioral responses, leading to outcomes uncoupled from in vitro binding capacity [73]. Fourth, functionally redundant OBPs with overlapping ligand spectra may collaboratively regulate the perception of a single volatile compound [74].

Adults of *R. cyatheae* feed on nectar and dew and lay eggs on the abaxial surface of young fronds of *A. spinulosa*. Larvae feed on young fronds, mature larvae pupate inside dry stems of host plants, and newly emerged adults continue to damage *A. spinulosa* [7], suggesting that OBP genes may be involved in host localization. The present study showed that RcyaOBP7 can bind in vitro to several ketone and alcohol volatiles, including *p*-ethylacetophenone, 2-octanone, (S)-3,3-dimethyl-2-butanol, and 2-ethyl-1-hexanol. Expression of RcyaOBP7 is higher in female antennae than in males, suggesting a possible role in host localization, but this requires in vivo functional validation. Fluorescence competitive binding assays verified only the in vitro binding capacity of RcyaOBP7 to VOCs, and molecular docking predicted intermolecular interactions and key binding residues. In vivo functional experiments (e.g., RNA interference or gene knockout) have not yet been conducted to clarify the specific role of this protein in antennal olfactory perception of *R. cyatheae*. Future in vivo studies are needed to elucidate the functional role of RcyaOBP7 in olfactory perception and to further understand the olfactory recognition process in this sawfly.

## 4. Materials and Methods

### 4.1. Insect Rearing and Tissue Collection

Test insects were collected from *R. cyatheae* habitats in the Guizhou Chishui Alsophila National Nature Reserve in China. during the active period. Test insects were collected as newly emerged adults (1–3 days post-emergence) and reared separately to ensure their virgin status. Larvae were field-collected and fed with tender fronds of *Alsophila* until reaching the mature instar, then transferred to rearing boxes in which *A. spinulosa* stems and fronds were placed to facilitate their eclosion. The rearing conditions were controlled as follows: temperature (25 ± 1) °C, relative humidity (RH) 75% ± 10%, and photoperiod 16L:8D.

### 4.2. VOCs Collection from Alsophila Plants and GC-EAD Analysis

Referring to the method described by Xu Tong [72], the dynamic headspace adsorption sampling procedure was slightly modified in this study. Volatile compounds from *A. spinulosa* were collected, and empty sampling bags without host plants were set as blank controls. The airflow rate was maintained at 500 mL/min using an atmospheric sampler (QC-1S, Beijing Ke’an Labor Protection New Technology Co., Ltd., Beijing, China). Sampling was conducted daily from 9:00 to 20:00, with a duration of 6 h for each sampling run. Three parallel replicates were arranged, and all replicate samples were pooled for elution for subsequent GC-MS and GC-EAD analyses. Intact whole *A. spinulosa* plants were placed into high-pressure sampling bags (40 cm × 60 cm). Two polytetrafluoroethylene (PTFE) tubes were inserted into the bag opening, which was tightly sealed with cable ties, serving as the air inlet and air outlet, respectively. The inlet of the sampling bag was connected to a purification system consisting of activated carbon, concentrated sulfuric acid solution and saturated sodium hydroxide solution in sequence, and then linked to the inlet of the atmospheric sampler through a flexible tube. The outlet of the sampling bag was connected to an adsorption tube, whose other end was connected to the outlet of the atmospheric sampler. All pipe joints in the device were connected with PTFE hoses to form a closed circulating gas circuit. After sampling, the adsorption tubes were eluted with 99.5% n-hexane. The eluates were transferred into 2 mL brown sample vials, which were sealed with Parafilm and stored at −20 °C in a refrigerator for later use.

A GC-EAD system was employed to identify the bioactive compounds in the VOCs of *A. spinulosa* that significantly influence the behavior of *R. cyatheae* (Nine post-eclosion, 1–3-day-old unmated adults underwent starvation pretreatment prior to testing.). Identical chromatographic parameters were applied to both GC-MS analysis and GC-FID detection combined with EAD measurement in this research. The volatiles of *A. spinulosa* were analyzed using a 7820A gas chromatograph (Agilent Technologies, Santa Clara, CA, USA), which was coupled with an electroantennographic detector (EAD; Syntech, Hilversum, The Netherlands). The EAD system comprised an IDAC4 signal acquisition controller, a CS-55 stimulus air controller, an MP-15 micromanipulator equipped with high-impedance PRG-3 electrodes, and an EC-03 gas chromatography split module heating system, where the PRG-3 electrodes were connected to the antennas via glass capillaries. EAG responses were measured using a Syntech instrument fitted with an IDAC-4 signal acquisition controller and a stimulus air controller. Data were recorded and analyzed using GC-EAD version 1.2.5 software (Syntech, Germany). After the gas chromatograph was stabilized, 3 μL of the concentrated plant extract was injected into the GC inlet, and the GC-EAD chromatograms were analyzed upon completion of the temperature program.

GC Operating Conditions: The chromatographic column was an HP-5 column (30 m × 0.32 mm × 0.25 μm, Agilent Technologies); the injector temperature was set at 250 °C with splitless injection, and the detector temperature was maintained at 300 °C. The column temperature program was as follows: held at 40 °C for 5 min, raised to 150 °C at a rate of 5 °C/min, then ramped to 280 °C at 10 °C/min, and held for 15 min. The temperature of the EC-03 module for EAD was set at 250 °C. The carrier gas was high-purity nitrogen, with a flow rate of 2.0 mL/min. All measurements were repeated three times for both male and female wasps. Chemical components were identified using the NIST Standard Database, and retention index (RI) were determined by comparison with experimental values in the NIST database. The relative contents of chemical components were calculated using the peak area normalization method [68]. By matching the retention order, peak shape, retention time, and mass spectral characteristics of the EAD-detected bioactive compounds with the corresponding data from GC-MS analysis, the specific components of these EAD-active compounds were finally identified.

### 4.3. The Electroantennographic (EAG) Response of A. spinulosa to Active VOCs

Adults of *R. cyatheae* used in this assay were 1–3 days post emergence, unmated, and subjected to starvation pretreatment before experiments. A total of 27 female and 27 male individuals were adopted for EAG detection, respectively. The antennal preparation method prior to the experiment was consistent with that used in the GC-EAD detection. Test compounds (Table 4) were diluted in n-hexane to prepare five concentration gradients: 0.01, 0.1, 1, 10, and 100 μL/mL. For each test, a 10 μL aliquot of the sample was applied to a clean filter paper strip of fixed dimensions (10 mm × 30 mm). The constant airflow was set at 1.0 L/min, and the stimulus airflow was set at 0.2 L/min. Each stimulus lasted for 0.5 s, with a 30-s interval between stimuli to ensure complete recovery of the antennal sensory activity. For the same antenna, an n-hexane control was performed first (EAG ck1), followed by sequential stimulation of the antenna with test compounds from low to high concentration, with each concentration applied once. A final n-hexane control was then conducted (EAG ck2). Each antenna was stimulated once per treatment, resulting in a total of seven stimulations per antenna. To establish the baseline value of EAG, measurements were taken with n-hexane both before and after the stimulation test, and the average value of these control measurements was used to calculate the relative EAG response. All samples were tested in triplicate to ensure data reliability. The calculation formula for the EAG response of each sample is as follows: continuous airflow

Relative EAG response = EAG − (EAG ck1 + EAG ck2)/2,(1)

where relative EAG response refers to the relative EAG response;

EAG ck1 represents the response amplitude (mV) of EAG to the test sample;

EAG ck2 denotes the response to the first and second control stimulations.

### 4.4. Behavioral Response of A. spinulosa to Active VOCs

Behavioral response assays using a Y-tube olfactometer were conducted with the bioactive volatile compounds that elicited significant EAG responses in the antennae of *R. cyatheae.* All standard compounds were prepared using n-hexane as the solvent at concentrations of 0.01, 0.1, 1, 10, and 100 μL/mL. For each trial, 100 μL of the test compound was pipetted onto a filter paper (ca. 24 cm^2^) and placed into an odor cartridge as the odor source. An equal volume of n-hexane was applied to another filter paper in a separate cartridge as the control. The airflow rate was adjusted to 0.8 L/min using a flow meter, and the ambient temperature was maintained at approximately 25 °C. Prior to the start of the assay, *R. cyatheae* were acclimated to the experimental environment for 5 h. Test sawflies were transferred to the inlet of the main arm of the Y-tube olfactometer. For each VOC, 30 male and 30 female *R. cyatheae* were tested, with 5 min of observation for each individual. The observation was carried out from 9:00 to 18:00. A blank control was performed before each treatment: sawflies were placed in the olfactometer with both arms supplied with filtered pure air, and their behavioral responses were observed to determine whether there was a selection bias. The Y-tube olfactometer consisted of a fully transparent Plexiglas Y-tube. A vacuum pump was used to drive air flow, which passed sequentially through activated carbon, an odor cartridge, a flow meter, and the Y-tube. A filter paper strip containing 100 μL of the volatile solution was placed on one arm of the Y-tube as the scented arm, while the unscented arm was fitted with a strip holding 100 μL of n-hexane as the control arm. For each sample, 30 males and 30 females were tested, with each individual subjected to only one test and not having been exposed to any volatile odorants prior to the assay. Each sawfly was observed for 5 min. A response was defined as a “choice” when a sawfly entered an arm and moved beyond the 1/3 length mark of the arm, remaining there for 1 min; otherwise, it was recorded as “no choice”. During the testing process, the Y-tube was replaced and the positions of the odor arm and odorless arm were alternated after every five insects to eliminate positional bias that could affect the *R. cyatheae*’s behavior. After each sample test, the odor cartridge and olfactometer were cleaned with 75% ethanol and distilled water, then dried thoroughly to remove any residual volatile odors before reuse.

### 4.5. Recombinant Expression and Purification of RcyaOBP7

One antennally enriched odorant-binding protein, RcyaOBP7, from *R. cyatheae* was selected to characterize its volatile-binding activity (Figure S2). The sequence of RcyaOBP7 was cloned into the pSmart-I vector and subsequently transformed into *E. coli* BL21 (DE3) competent cells. After confirming target protein expression via SDS-PAGE and Western blot, the recombinant strains were inoculated into 1 L LB medium for large-scale cultivation at 37 °C with a shaking speed of 220 rpm. When the OD_600_ value of the bacterial culture reached 0.6–0.8, IPTG at the optimized concentration was added, and induced expression was carried out at 15 °C for 16 h to produce the target protein. The bacterial cells were collected by centrifugation at 4 °C, resuspended in Tris-NaCl buffer, and lysed by sonication. The supernatant was collected for subsequent purification. The target protein was purified by two-step Ni-NTA affinity chromatography. For the first purification step, equilibration buffer (PBS-NaCl, pH 7.4), washing buffer (PBS-NaCl + 50 mM imidazole, pH 7.4), and elution buffer (PBS-NaCl + 500 mM imidazole, pH 7.4) were used; the eluted samples were dialyzed to remove imidazole, followed by digestion with SUMO protease and incubation overnight at 4 °C, then subjected to the second round of Ni-NTA affinity chromatography using the same buffer system. Purification efficiency was analyzed by SDS-PAGE. The purified protein was dialyzed into PBS buffer (containing 300 mM NaCl and 10% glycerol, pH 7.4), then concentrated and sterile-filtered. Protein concentration was determined using the Bradford method [75,76].

### 4.6. Fluorescence Competitive Binding Assay

The binding affinity between RcyaOBP7 and candidate ligands was determined using a multimode microplate reader (BioTek Synergy H1, Agilent BioTek, Winooski, VT, USA). Nine electrophysiologically active compounds (screened via GC-EAD to elicit antennal responses in *R. cyatheae*) were selected for the assay (Table 3). The fluorescent probe N-phenyl-1-naphthylamine (1-NPN) and all ligands were dissolved in chromatographic-grade methanol to a final concentration of 1 mM. To characterize the binding of 1-NPN to RcyaOBP7, titration was performed by adding 1 mM 1-NPN to a 2 μM purified protein solution (10 mM PBS, pH 7.4), resulting in a final 1-NPN concentration range of 1–28 μM. The excitation and emission wavelengths were set at 337 nm and 450 nm, respectively. The dissociation constant (Kd) of 1-NPN with RcyaOBP7 was calculated using the Scatchard equation [77]. In ligand-binding experiments, each ligand was added to a mixture containing 2 μM purified protein and 2 μM 1-NPN, resulting in final ligand concentrations ranging from 1 to 40 μM. Each ligand assay was performed in triplicate. The dissociation constant (Ki) of RcyaOBP7 with ligands was calculated using the formula: Ki= [IC50]/(1 + [1 − NPN]/K1 − NPN) where [IC50] = ligand concentration required to reduce the initial fluorescence by 50%, [1 − NPN] = free concentration of 1− NPN, and K1 − NPN = dissociation constant of the RcyaOBP7/1− NPN complex [78]. Ligands with a Ki value < 22 μM were defined as having “strong” binding affinity for RcyaOBP7, while those with Ki values of 22–40 μM exhibited “moderate” binding affinity [23].

### 4.7. Structural Modeling and Molecular Docking

The amino acid sequence of RcyaOBP7 was submitted to the Robetta web server (https://robetta.bakerlab.org/ (accessed on 10 March 2026)) for three-dimensional structure modeling [79]. The quality of the generated 3D structure was evaluated using the Structure Analysis and Verification Server (SAVES v6.1, https://saves.mbi.ucla.edu/ (accessed on 10 March 2026)). For these ligands, their three-dimensional structures were initially retrieved from the PubChem database (https://pubchem.ncbi.nlm.nih.gov/ (accessed on 10 March 2026)) and then converted to PDB format using Open Babel GUI version 3.1.1 [80]. Molecular docking calculations between RcyaOBP7 and the nine ligands were performed by following the standard protocol in AutoDockTools 1.5.6. Finally, the molecular docking results were visualized and analyzed using PyMOL v3.0.3. Non-covalent interactions between the protein and ligands were identified and visualized using PLIP (https://plip-tool.biotec.tu-dresden.de/plip-web/plip/index (accessed on 10 March 2026)).

### 4.8. Data Analysis

Mass spectral data were matched against the NIST standard library for the qualitative and quantitative analysis of *A spinulosa* volatiles. All other experimental data were analyzed using SPSS 25.0, GraphPad Prism 9.5.0, and Origin 2021 software. Specifically, differences in the Y-tube olfactometer bioassay data were determined via the chi-square (χ^2^) test. For the evaluation of the effects of concentration gradient, sexual dimorphism, and VOC species, as well as their interactions, two-way analysis of variance (two-way ANOVA) was performed using GraphPad Prism 9.5.0, followed by Tukey’s honestly significant difference (HSD) test for multiple comparisons. Differences were considered statistically significant at *p* < 0.05. All data are presented as the mean ± standard error (SE) of at least three independent replicates.

## 5. Conclusions

Host plant volatiles can regulate insect behavior, and insects utilize plant volatiles as cues for host localization and selection. The present study confirmed that VOCs from the host plant *A. spinulosa* exert a selective regulatory effect on the olfactory-driven host location behavior of *R. cyatheae*. In vitro binding assays showed that RcyaOBP7 binds specifically to *p*-ethylacetophenone, suggesting a potential role that requires in vivo validation in host plant volatile recognition. Our results describe the VOC profile of *A. spinulosa* and the behavioral responses of *R. cyatheae* to these volatiles. While RcyaOBP7 and *p*-ethylacetophenone show promise as research targets for pest management, their in vivo relevance remains to be demonstrated. Therefore, any practical applications would require further validation, including functional studies in vivo (e.g., gene silencing), examination of other OBPs and olfactory receptors, and field-based behavioral assays under ecologically relevant conditions.

## Figures and Tables

**Table 1:** Volatile organic compounds detected from *A. spinulosa* by GC-MS method.

No.	RT	VOCs	RI (HP-5)	Contents (%)
1	3.74	3-buten-2-ol	665	0.05%
2	3.93	3,3-dimethyl-2-butanol	768	1.16%
3	4.12	3-ethyl-3-hexene	780	0.29%
4	4.65	2-methylheptane	770	0.63%
5	4.90	3-methylheptane	774	4.06%
6	5.03	2-methyl-1-butanol,	825	0.02%
7	5.55	5-methyl-3-heptene	805	1.86%
8	5.82	3-ethyl-2-hexene	795	6.64%
9	5.94	2-methyl-2-heptene	788	4.30%
10	6.09	2-octene	800	2.14%
11	7.24	pyruvic acid, butyl ester	920	0.38%
12	7.61	2-butanone	620	0.06%
13	8.30	2,5-diethyltetrahydro-Furan	890	0.40%
14	8.42	1,3-dimethyl-benzene	872	0.08%
15	9.35	*p*-xylene	868	0.41%
16	9.68	3,3-dimethyl-2-hexanone	915	0.65%
17	11.35	3-methyl-2-heptanone	930	2.72%
18	12.59	3-methyl-2-heptanol	975	0.08%
19	11.77	2-methyl-pentanal	835	0.43%
20	12.08	2-methyl-3-heptanol	970	0.67%
21	12.71	hexyl lactate	1170	0.57%
22	13.07	2-octanone	985	1.43%
23	13.25	3-octanol	990	0.78%
24	13.36	decane	1000	0.83%
25	13.44	(S)-2-hexanol	870	0.38%
26	14.41	2-ethyl-1-hexanol	1040	24.60%
27	16.73	undecane	1100	2.06%
28	16.87	cis-3-hexenyl crotonate	1280	0.59%
29	19.77	vinyl trans-cinnamate	1420	0.02%
30	19.81	dodecane	1200	2.43%
31	20.35	1,1-dimethyl-1H-Indene	1210	0.04%
32	21.42	thymol	1290	5.29%
33	21.54	nonanal	1104	0.01%
34	21.67	*p*-ethylacetophenone	1450	22.74%
35	22.61	tridecane	1300	6.51%
36	25.33	tetradecane	1400	1.89%
37	25.78	cedrene	1490	0.58%
38	26.67	3,3-dimethylhexane	860	1.89%
39	29.70	2,4-dimethyl-heptane	875	0.32%

**Table 2:** Thirteen EAD-active VOCs were identified from *A. spinulosa*.

No.	VOCs	Retention Time (min)
1	(S)-3,3-dimethyl-2-butanol	3.93
2	3-methylheptane	4.90
3	5-methyl-3-heptene	5.55
4	2-methyl-2-heptene	5.94
5	*p*-xylene	9.35
6	3-methyl-2-heptanone	11.35
7	3-methyl-2-heptanol	12.59
8	2-octanone	13.07
9	2-ethyl-1-hexanol	14.41
10	*p*-ethylacetophenone	21.67
11	tridecane	22.61
12	tetradecane	25.33
13	cedrene	25.78

**Table 4:** Standard active compounds for test.

No	CAS	Vendors	Molecular Formulate
1	937-30-4	Yuanye	*p*-ethylacetophenone
2	106-42-3	Macklin	*p*-xylene
3	1517-67-5	KaiweiChemical	(S)-3,3-dimethyl-2-butanol
4	589-81-1	Macklin	3-methylheptane
5	111-13-7	Macklin	2-octanone
6	11028-42-5	Yuanye	cedrene
7	629-50-5	Macklin	tridecane
8	629-59-4	Macklin	tetradecane
9	104-76-7	Macklin	2-ethyl-1-hexanol

## Data Availability

All data generated or analyzed during this study are included in this published article and its Supplementary Information Files. The transcriptome data supporting the analysis of RcyaOBP7 have been submitted and are available from the corresponding author upon reasonable request.

## Supplementary Materials

The following supporting information can be downloaded at: https://www.mdpi.com/article/10.3390/ijms27094029/s1.

## Abbreviations

The following abbreviations are used in this manuscript:

VOCs	Volatile organic compounds
GC-MS	Gas chromatography-mass spectrometry
GC-EAD	Gas chromatography-electroantennography
OBPs	Odorant-binding proteins
CSPs	Chemosensory proteins
NPC2s	Niemann-Pick type C2 proteins
ORs	Odorant receptors
GRs	Gustatory receptors
IRs	Ionotropic receptors
SNMPs	Sensory neuron membrane proteins
ORNs	Olfactory receptor neurons
