# Supplementary material for: Antennal Responses and Odorant-Binding Protein 7 Binding of Rhoptroceros cyatheae (Selandriidae: Rhopographus) to Volatile Organic Compounds from Alsophila spinulosa
Source: Int J Mol Sci. 2026 Apr 30;27(9):4029. doi: 10.3390/ijms27094029 (PMC13164018; doi:10.3390/ijms27094029)
Supplement: Supplementary file 1 [file ijms-27-04029-s001.zip › ijms-4223106-supplementary-main.pdf]

Supplementary Materials

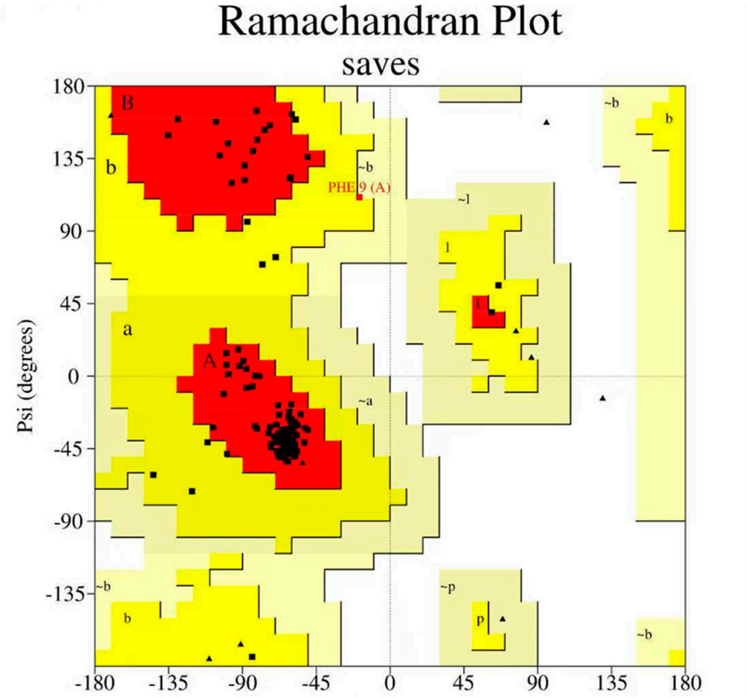

Figure S1. Ramachandran plot of the structure of RcyaOBP7

Note: The disallowed region (white); the corresponding region (light yellow), including a, b, l, p areas; the best region (Red), including A, B, L areas; barely permissible range (pale yellow), including ~a, ~b, ~l, ~p areas.

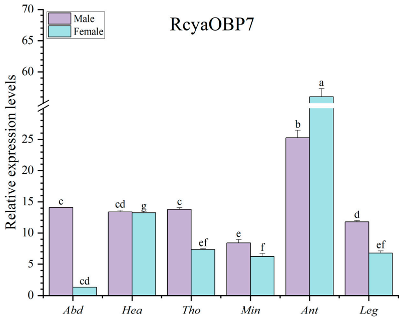

Figure S2. Expression profiles of the *RcyaOBP7* genes in different *R. cyatheae* 12 tissues (submitted for publication).

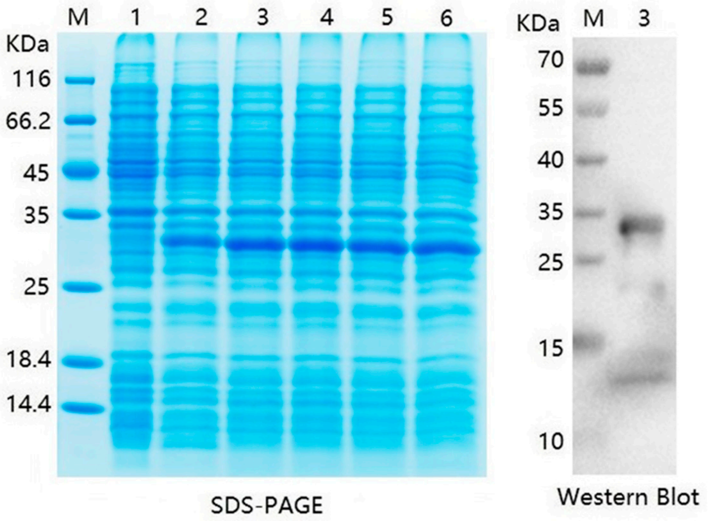

Figure S3. Expression identification results of SDS-PAGE and Western Blot

Note: Lane M, Protein Marker; Lane 1, uninduced sample; Lanes 2–6, induced samples.

Table S1. The relative EAG response values of *Rhoptroceros cyatheae*.

Relative EAG Response Values (rEAG)										
**VOCs**	**F**	**M**	**F**	**M**	**F**	**M**	**F**	**M**	**F**	**M**
	**0.01μL/mL**	**0.01μL/mL**	**0.1 μL/mL**	**0.1 μL/mL**	**1μL/mL**	**1μL/mL**	**10μL/mL**	**10μL/mL**	**100 μL/mL**	**100 μL/mL**
***p*-xylene**	0.009±0.022a	0.008±0.001d	0.031±0.044cd	0.004±0.005d	0.063±0.048c	0.022±0.011cd	0.348±0.174b	0.232±0.034b	0.613±0.043a	0.735±0.077a
**cedrene**	0.003±0.01b	0.003±0.009b	0.004±0.012b	0.006±0.008b	0.023±0.019ab	0.032±0.013ab	0.116±0.017a	0.143±0.028a	0.121±0.018a	0.15±0.026
**3-methylheptane**	0.099±0.067a	0.002±0.002a	0.094±0.072a	0.002±0.002a	0.105±0.076a	0.009±0.004a	0.127±0.079a	0.015±0.003a	0.163±0.063a	0.159±0.012a
**(S)-3,3-dimethyl-2-butanol**	0.01±0.007d	0.005±0.004d	0.012±0.006c	0.021±0.017bc	0.013±0.019c	0.117±0.119c	0.066±0.045b	0.289±0.292b	0.945±0.033a	0.835±0.243a
***p*-ethylacetophenone**	0.035±0.062cd	0.003±0.002d	0.058±0.054cd	0.047±0.007cd	0.197±0.054c	0.293±0.037cb	0.425±0.02b	0.746±0.051b	0.432±0.024a	0.754±0.06a
**2-octanone**	0.008±0.107e	0.005±0.007e	0.019±0.101de	0.006±0.011e	0.048±0.109d	0.123±0.039cd	0.224±0.17c	0.553±0.069ab	0.447±0.132b	0.729±0.066a
**tridecane**	0.006±0.006cd	0±0.002cd	0.01±0.004c	0.003±0.004cd	0.045±0.006b	0.016±0.015bc	0.235±0.02a	0.108±0.017b	0.243±0.022a	0.12±0.016bc
**tetradecane**	0.014±0.038ab	0.002±0.018ab	0.033±0.05ab	0.007±0.02ab	0.034±0.051a	0.004±0.019ab	0.044±0.061a	0.135±0.023a	0.17±0.107a	0.146±0.028a
**2-ethyl-1-hexanol,**	0.005±0.015cd	0.001±0.004cd	0.045±0.02c	0.048±0.009c	0.299±0.063b	0.305±0.04b	0.649±0.077a	0.718±0.072a	0.668±0.068a	0.731±0.085a

Nucleotide sequence of the RcyaOBP7 gene: CAAACCTCTCTGAAAAATCATGAATCTTTCAATCGCCTTTATTTTCGGGCT
CGTCTGCCTAACGGCAGCTCTCGCTCACAAGGGACTGACACCGGAACAAATAGCGAAGATGAAAGAGATGAAGGAGAA
GTGCGCGACGGAAACCGGTGTAGATGTCGAAGTGTTGAAAAATCCCGGTGGAGAAGAAGCGATGAAGGACGAAAAGCT
GCATTGTTTTGGAGCTTGCATTCTAAAAACTTTGGAAATCCTCGACGAGGATGGAAATTTCAACAAGGACGTTGCAATTGA
AAAAATACCTGATGGTTTACCGAAAGACAAGATGACGGAGATCATAAATGATTGTGCAAAAGAGAAAGGCGCAACACCT
TGCGAAACGGCTAAAATTCTATTCACGTGTTTCCACGAGCATAAGGATCACGAAGTCATGGAGATGCTGCATCATTGATGG
CTGTCAAAGAAACGGAATCAATCAACGAGAAATATTTCAACTATAAGATAAAAATGAATTTATAATGAATTATTTGAATA
TGAATATTTCTCTTTACAAGCTTAAGTTCTTCAATTCTATTCGCAAATTAAAATGATGTATATACGTATCAATGGGCGTGAA
TTTTTTTTTTATCCTCGTACTTGTCGTTCTCCGGATTGACACCGAATTGCTTTTTATTAACAAAAAAAAAAAAAAAAAACAAA
ATCCTTTACGTGCACAAATTTCTGTGACTATAACAGCGATGGAGTTTACTTTAGGCAAATTAATAACGTCAAAGATGAAGT
TTGTCTTTAAAATTTAAACGTCGAATGATGTATCGTTCGTGAACTTTGTTAACAGCGTTCAGACCAGCAAATGAACGTTGCC
